# Sleep quality and duration and frailty in older adults: a systematic review

**DOI:** 10.3389/fpubh.2025.1539849

**Published:** 2025-02-26

**Authors:** Ângela Maria Natal de Souza, Dalila Pinto de Souza Fernandes, Isabella Silva Castro, Fernanda Gaspar Gróla, Andréia Queiroz Ribeiro

**Affiliations:** ^1^Department of Nutrition and Health, Federal University of Viçosa, Viçosa, Minas Gerais, Brazil; ^2^University Restaurant, Federal University of Juiz de Fora, Juiz de Fora, Minas Gerais, Brazil; ^3^School of Nursing, Federal University of Minas Gerais, Belo Horizonte, Minas Gerais, Brazil

**Keywords:** older adults, frailty, sleep quality, sleep complaints, aging, systematic review

## Abstract

**Introduction:**

Sleep problems and frailty are associated with adverse health outcomes in older adults, including mortality, and constitute a major public health challenge.

**Objective:**

This study investigated the association between sleep quality and duration and frailty in older adults, with emphasis on methods of evaluation.

**Methods:**

This systematic review was guided by the Preferred Reporting Items for Systematic Reviews and MetaAnalyses (PRISMA). The Embase, Medline (Pubmed) and Cochrane libraries were searched, with no time restrictions for publications.

**Results and discussion:**

Of the 17 studies included in this review, all published between 2009 and 2024, 13 were cross-sectional and only four were longitudinal. The Pittsburgh Sleep Quality Index and the Fried phenotype were widely used as methods to assess, respectively, sleep and frailty. Studies evaluating insomnia and frailty by the Fried phenotype all found an independent association. Poor sleep quality was independently associated with pre-frailty and frailty. Sleep onset insomnia, but not sleep maintenance insomnia, was associated with frailty. Short (5 h) and long (9 h) sleep duration were also associated with frailty. Poor sleep quality was associated with pre-frailty and frailty in older adults. The results show a wide diversity of methods for assessing both exposure (sleep quality) and outcome (frailty) and point to a need for further – especially longitudinal – research on the relationship between sleep and frailty.

## 1 Introduction

Sleep is crucial to health, since during this process a number of reactions take place to maintain body homeostasis (1). Sleep-related problems are highly prevalent worldwide. Insomnia is defined as a sleep disorder characterized by dissatisfaction with qualitative or quantitative sleep (2), especially, is between 12 and 20% prevalent in older adults (2) and a challenge for public health, given the risks it poses for healthy aging. Sleep problems and disorders are associated with higher risks of mortality, cardiovascular diseases, depression and other conditions (3–5).

Meanwhile, another challenge to healthy aging is frailty, a multidimensional condition that leads to a decline in the ability to resist internal and external stressors, thus increasing both harmful effects on various organs and individual vulnerability (6) and associated with higher risk of hospitalization, falls and mortality (7, 8).

There has been growing interest in investigating the association between sleep quality and duration and frailty in older adults, because both conditions are precursors of disability and morbimortality (9). However, studies of this relationship have returned inconsistent results. Some studies find an association between only short (< 5 h) sleep duration and frailty (10, 11), while others find an association with only long (> 9 h) sleep duration (12) and others observe frailty to be associated with both short and long sleep duration (11).

In addition, methods of measuring both sleep and frailty vary considerably, and this may be one of the reasons for inconsistent results. Sleep can be evaluated by using subjective and objective measures (13), and studies that combine both can provide more comprehensive information about sleep quality and duration. Objective measurement is particularly important in studies of populations of older adults, because of the cognitive decline, including difficulties with memory, common in this age group (14). Similarly, various tools are used to assess frailty, chiefly the frailty phenotype (7), frailty index (15) and frailty scale (16).

Accordingly, this systematic review investigated the association between sleep quality and duration and frailty in non-institutionalized older adults, with emphasis on the methods employed in the studies to evaluate these events.

## 2 Methods

This systematic review was conducted according to the Preferred Reporting Items for Systematic Reviews and Meta-Analyses (PRISMA) (17) and registered in the International Prospective Register of Systematic Reviews (PROSPERO) under No. CD42023441262.

The research question, “Is there an association between sleep quality and frailty in older adults?,” applied the following PICOS strategy:

P (population): community-dwelling older adults over 60 years of age.

I (exposure): sleep quality as diagnosed by the criteria used in the original studies.

C (control): does not apply.

O (outcome): frailty as diagnosed by the criteria used in the original studies.

S (study design): observational studies.

### 2.1 Search strategy

Studies were retrieved by searching the Pubmed, Embase and Cochrane Library electronic databases, using a combination of MESH search terms (“sleep quality” OR “sleep hygiene”) AND (frailty OR “frail older adults” OR “frailty syndrome” OR frailties) and EMTREE terms (“sleep quality” OR “sleep quality scale” OR “sleep disorders”) AND (Frailty OR “Frailty phenotype” OR “Frailty Syndrome” OR fragility). The reference lists of the relevant original and review articles were also screened manually for related studies. The searches were performed independently by two authors (AMNS; DPSF), the last in August 2024.

### 2.2 Criteria for inclusion and exclusion

#### 2.2.1 Inclusion criteria

Original observational (longitudinal and cross-sectional) studies of older adults (60 years of age or older), published in English, Spanish and Portuguese, were included, irrespective of publication date.

#### 2.2.2 Exclusion criteria

**Figure 1:**
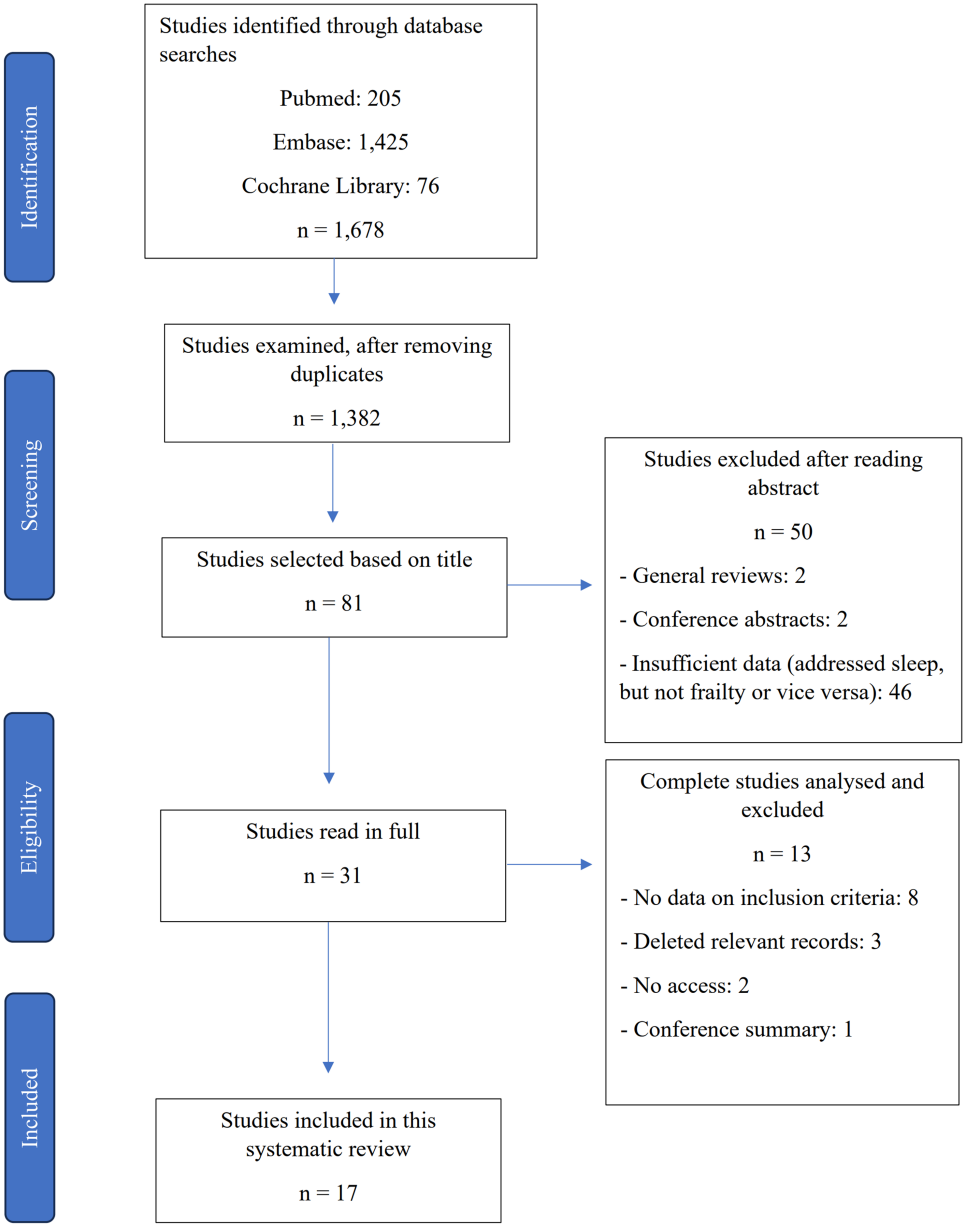
Flowchart of study inclusion and exclusion criteria.

Animal studies, studies whose abstract and full-length article could not be accessed, studies of elders living in nursing homes for older adults and studies formatted as posters, letters of comment, conference proceedings, summaries and reviews were not included. Lastly, studies of populations with specific conditions were also not included (Figure 1).

### 2.3 Selection of studies

Two authors searched each database independently, using the specified search strategies. First, articles were selected by title and then, by reading the abstract. Duplicate articles were removed and a final selection was made by reading in full against the inclusion criteria. In the event of disagreements, a third researcher (AQR) was called on.

### 2.4 Data extraction

Once articles had been read and approved for inclusion in the systematic review, the following data were extracted: author, year and country of publication, study population, study type, sleep assessment methods, frailty assessment methods and main results. Data were also extracted by two authors independently and then compared.

### 2.5 Quality assessment

Studies included in this review were assessed for methodological quality using the Checklist proposed by the Joanna Briggs Institute (18), which is specific to each study type (cohort, case–control, cross-sectional and so on). After evaluation, the studies were classified as low (< 50%), moderate (50–70%) or high (≥ 70%) quality (19) (Supplementary Tables S1, S2).

## 3 Results

The 17 studies included (Figure 1) evaluated a total of 58,447 older adults, whose average age ranged from 64 to 84 years. Most studies were cross-sectional and only four were prospective cohorts (20–23). In 10 studies, the population was mostly female. Most of the studies were conducted in China (12, 23–26), followed by Mexico (10, 11, 27) and the United States (20, 28, 29) (Table 1). No studies were retrieved by the reverse search.

**Table 1:** Main characteristics of the studies included in this review.

Author, Year and Country	Study population	Type of study	Sleep assessment	Frailty assessment	Adjustment variables	Main results
Vaz-Fragoso et al., 2009 (28) United States	384 participants Mean age: 84.3 ± 4.5 Female, 67.4%	Cross-sectional	Epworth Sleepiness Scale Insomnia Severity Index	Frailty Phenotype Criteria similar to Fried’s criteria	Age, sex, number of chronic conditions, number of medications, use of medications with adverse effects on the central nervous system, health status, cognitive impairment, depressive symptoms.	Frailty, 4.2%. Significant association between sleepiness and frailty in adjusted models (OR = 3.67; 95% CI = 2.03–6.61). Clinical insomnia was significantly associated with frailty in the gross analysis (OR = 2.77, 95% CI = 1.36–5.67), but not in the adjusted analysis (OR = 1.93, 95% CI = 0.81–4.61).
Ensrud et al., 2009 (29) United States	3,133 participants 67 years old or more Male, 100%	Cross-sectional	Pittsburgh Sleep Quality Index (PSQI) Epworth Sleepiness Scale Actigraphy Polysomnography	Frailty Phenotype- Criteria similar to Fried’s criteria	Age, sex, location, number of medical conditions, body mass index, social support, educational level, alcohol consumption, smoking, antidepressant use, non-barbiturate benzodiazepine use, number of medical conditions, depressive symptoms, functional impairments.	Frailty, 14%. After adjustments: Poor subjective sleep quality (PSQI >5) (OR = 1.28; 95% CI = 1.09–1.50) was associated with an increase in the chances of a greater state of frailty. Both reduced sleep efficiency (OR = 1.37; 95% CI = 1.12–1.67) and prolonged sleep latency (OR = 1.42; 95% CI = 1.10–1.82) were independently associated with frailty, as were respiratory disorders (OR = 1.38, 95% CI = 1.15–1.65).
Ensrud et al., 2012 (20) United States	2,505 participants 67 years old or more Male, 100%	Prospective cohort 3.4 years of follow-up	Pittsburgh Sleep Quality Index (PSQI) Epworth Sleepiness Scale Actigraphy Polysomnography	Frailty Phenotype- Criteria similar to Fried’s criteria	Age, sex, location, number of medical conditions, body mass index, social support, educational level, alcohol consumption, smoking, antidepressant use, non-barbiturate benzodiazepine use, number of medical conditions, baseline frailty status (robust stage or intermediary).	Incidence - Frailty, 14.4%; pre- frailty 50.5%. Sleep duration (OR = 1.15; 95% CI = 0.82–1.62) and prolonged sleep latency (OR = 1.20; 95% CI = 0.84–1.71) were not associated with frailty and mortality during follow-up time. Poor subjective sleep quality (multivariable odds ratio [MOR] 1.26, 95% CI = 1.01–1.58), greater nighttime wakefulness (MOR 1.31, 95% CI = 1.04–1.66), and greater nocturnal hypoxemia (MOR 1.47, 95% CI = 1.02–2.10) were associated with a higher odds of frailty/death at follow-up (vs. robust/intermediate), greater nighttime wakefulness (MOR 1.57, 95% CI = 1.12–2.20) were associated with a higher odds of death
Moreno-Tomayo et al., 2017 (27) Mexico	521 older adults Mean age:76.3 ± 3.3 Female 52.8%	Cross-sectional	Self-reported sleep question *Have you had sleeping problems recently?* yes or no	Frailty Phenotype	Age, sex, literacy, ethnicity, work situation, marital status, difficulties in basic activities of daily living, difficulties in instrumental activities.	Frailty, 10.7%; Pre-frailty, 51.9%; Sleep complaints, 20.0%. Sleep complaints in women were associated with frailty in rural older adults (OR = 3.20; 95% CI = 1.33–7.68). For men there was no association (OR = 0.76; CI% = 0.23–2.51).
Sun et al., 2020 (12) China	1726 older adults Mean age: 77.7 ± 3.9 Female, 52.7%	Cross-sectional	Pittsburgh Sleep Quality Index	Frailty Phenotype (adapted)	Age, sex, smoking, alcohol consumption, education, marital status, occupation, body mass index, diabetes, hypertension, health perception, sleep quality.	Frailty, 9.2%; Pre- frailty, 53.8%; poor sleep quality, 43.6%. Long sleep duration (≥ 9 h) was associated with an increased risk of pre-frailty (OR = 1.96; 95% CI = 1.51–2.53) and frailty (OR = 2.55; 95% CI = 1.49–4.35) in adjusted models. Poor subjective sleep quality was associated with pre-frailty in the crude model (OR = 1.75; 95% CI = 1.43–2.16) and in the adjusted model (OR = 1.51; 95% CI = 1.20–1.90).
Shih et al., 2020 (24) China	828 older adults Mean age: 64 years old Female, 71.4%	Cross-sectional	Pittsburgh Sleep Quality Index	Frailty Phenotype	Age, sex, body mass index, appendicular skeletal muscle index, handgrip strength, exercise habit, gait speed.	Pre-frailty and frailty, 28.2%. Poor sleep quality (IQSP >5) was associated with frailty in older adults (OR = 1.95; 95% CI = 1.38–2.77).
Moreno-Tamayo et al., 2020 (10) Mexico	493 older adults Mean age:70.1 ± 5.6 Female, 60.7%	Cross-sectional	Athens Insomnia Scale Pittsburgh Sleep Quality Index	Frailty Phenotype	Age, sex, marital status, cognitive decline, depression, limitations in daily activities, polypharmacy, current smoking, alcohol consumption and comorbidities.	Frailty 13.4%; pre-frailty, 24.1%; In models adjusted for insomnia (OR = 3.23; 95% CI = 1.48–7.07); low sleep quality (OR = 3.34; 95% CI = 1.37–8.15) and sleeping less than 5 hours (OR = 2.67; 95% CI = 1.01–7.08) were shown to increase the chances of frailty in females.
Nemoto et al., 2021 (21) Japan	3,844 older adults Mean age:74 ± 6.4 Female, 55.3%	Cohort 2 years of follow-up	Athens Insomnia Scale	Kihon Checklist - self-administered questionnaire	Age, sex, education, professional status, self-rated health, comorbidities, body mass index, physical activity, alcohol consumption, smoking.	Incidence of frailty 30.0%; pre- frailty 30.4% The cross-lagged panel model was used. Frailty was associated with insomnia (standardized coefficient [95% CI]: 0.076 [0.045–0.107]) and insomnia was also associated with frailty (0.074 [0.044–0.104]). In older men, the standardized coefficient (95% CI) was 0.087 (0.039, 0.135) for frailty on insomnia and 0.051 (0.005, 0.096) for insomnia on frailty. In older women, the standardized coefficient (95% CI) was 0.067 (0.022, 0.112) for the cross-lagged association of frailty on insomnia, and 0.100 (0.059, 0.141) for insomnia on frailty
Balomenos et al., 2021(32) Greece	1984 older adults Mean age:73.9 ± 5.4 Female, 59.2%	Cross-sectional	Sleep scale	Frailty Index, Tilburg Frailty Indicator and Groningen Frailty Indicator	Age, sex, education, sleep quality, sleep duration, use of sleep-related medications.	Frailty 19.6% (FI); 31.2% (TFI); 30.6% (GFI). Low sleep quality was associated with frailty assessed by the FI (OR = 3.76 95% CI = 2.68–5.29); TFI (OR = 2.68; 95% CI = 1.99–3.61); IFG (OR = 2.55 95% CI = 1.88–3.46). Participants with long sleep duration were more likely to be frail according to FI (OR = 1.61; 95% CI = 1.03–2.53) and GFI (OR = 1.86; 95% CI = 1.20–2.90).
Alqahtani, 2021(30) Saudi Arabia	270 older adults Mean age: 69 years old Male, 65.2%	Cross-sectional	Pittsburgh Sleep Quality Index	Frailty Phenotype	Age, sex, body mass index, handgrip strength, living situation, marital status, gait speed, Mini Mental Health Examination score.	Frailty 29.2%; Pre- frailty, 37.7%; Poor sleep quality, 65.9%; Poor sleep quality was independently associated with pre-frailty (OR: 1.67; 95% CI = 1.26–2.05) and frailty (OR: 2.13; 95% CI = 1.47–3.12) after adjustment for potential confounding factors.
Moreno-Tomayo et al., 2021(22) Mexico	309 older adults Mean age: 76.2 years old Female, 55.3%	Prospective cohort 5 years of follow-up	Sleep duration: self-reported questions Sleep complaints: (*Have you had any problems sleeping recently?*)	Frailty Phenotype	Age, sex, ethnic origin, literacy, paid work, have a partner, subjective memory complaints, difficulties in basic activities of daily living, difficulties in instrumental activities, number of medications, presence of hypertension, diabetes, hypercholesterolemia, coronary heart disease, heart attack, arthritis and osteoporosis.	The cumulative incidence of frailty was 30.4%. Among older adults short sleep duration ≤5 h, the RR was 1.80 (95% CI = 1.04–3.11) and among those long duration 9 h or more, the RR was 1.69 (95% CI = 1.10–2.58) compared to those sleep time 7-8 h per night. Sleep complaints were not associated with the incidence of frailty (RR = 1.41; 95% CI = 0.94–2.12).
Moreno-Tamayo et al., 2021(11) Mexico	1,643 older adults Mean age: 67.1 ± 5.9 Male, 50.1%	Cross-sectional	Berlin Questionnaire Athens Insomnia Scale Epworth Sleepiness Scale	Frailty Phenotype	Age, sex, ethnic origin, literacy, paid work, have a partner, subjective memory complaints, difficulties in basic activities of daily living, difficulties in instrumental activities, number of medications, presence of hypertension, diabetes, hypercholesterolemia, coronary heart disease, heart attack, arthritis and osteoporosis, Short Physical Performance Battery score.	Frailty, 10.5%. In both males and females, the high risk of OSA (OR 1.59; 95% CI = 1.07–2.38) and insomnia (OR 2.11; 95% CI = 1.41–3.16) was associated with fragility. Insomnia was associated with frailty in males (OR 1.88; 95% CI = 1.01–3.52) and females (OR = 2.38; 95% CI = 1.35–4.20). In females, the high risk of OSA (OR = 1.84; 95% CI = 1.05–3.22) and insomnia (OR 2.38; 95% CI = 1.35–4.20) were associated with frailty.
Yu et al., 2022(25) China	2,647 older adults Mean age: 68.59 ± 6.13 Female, 58.9%	Cross-sectional	Pittsburgh Sleep Quality Index	Assessment Instrument Comprehensive Fragility	Not applicable	Mild frailty (55.8%); high frailty (14.8%). Poor sleep quality (>7) is associated with mild frailty OR 1.57 (95% CI = 1.22–2.03). Poor sleep quality (>7) is associated with high frailty (OR = 2.28 95% CI = 1.64–3.17).
Fan et al., 2022(26) China	454 older adults No further information available Female, 57.7%	Cross-sectional	Athens Insomnia Scale	Tilburg Fragility Indicator - Chinese version	Information not available	Frailty 40.7%. Insomnia was independently associated with multidimensional frailty after adjustments (OR = 6.86; 95% CI = 4.237–11.116).
Chen et al., 2022(23) China	7,623 older adults Mean 84 years old Female, 51.5% Residents in rural areas, 65.8%	Retrospective cohort 4.4 years of follow-up	Self-reported question *“Normally, how many hours do you sleep?”*	Frailty Index	Age, sex, cognitive function, location, living status, having your own room, self-rated financial situation, body mass index, alcohol consumption, smoking, fruit consumption, vegetable consumption, physical activity index and sleep quality.	Incidence of frailty 33.2%; 25.1% participants with long sleep duration. Those with long sleep duration have a higher risk of frailty [1.20 (95% CI = 1.05–1.37)] compared to those with medium and short sleep duration.
Aditi et al., 2023(9) India	31,902 older adults (15,139 males and 16,763 females)	Cross-sectional	Questions - Jenkins Sleep Scale (JSS-4) (1) How often do you have trouble falling asleep? (2) How often did you wake up too early in the morning and were not being able to fall asleep again? (3) How often did you wake up during the night and have trouble getting back to sleep? The report occasionally and frequently to any of the questions was considered as sleep disorder (mainly insomnia).	Frailty Phenotype	Not applicable	Frailty: 21.3% 32 and 40% of older men and women had sleep disorders, respectively. Older adults with a sleep disorder were 1.66 times more likely (95% CI = 1.47—1.87) to suffer from frailty compared to those without a sleep disorder.
Mizuno et al. 2023(31) Japan	866 participants Aged in their 70s and 80s Female, 50.8%	Cross-sectional	Japanese version of the Pittsburgh Sleep Quality Index (PSQI) to assess sleep quality (PSQI global score) and sleep medication use (PSQI component 6)	Japanese version of the Cardiovascular Health Study criteria based on the phenotype of frailty, which has validity and standardized definition (7, 43)	Sex, BMI, education, cognitive function, mental health, lifestyle habits (smoking and drinking), hypertension, and diabetes, as well as histories of stroke, heart disease, and joint disease (31).	Frailty:19.7%; Short sleep (<6 h): 13.4%; Long sleep (>8 h):12.5%; Poor sleep quality: 42.1% In the 80-year-old age group, poor sleep quality (OR = 1.78, 95% CI = [1.02, 3.11]) was independently associated with frailty.

h, hour; FI, fragility index; TFI, Tilburg fragility indicator; GFI, Groningen frailty indicator; BMI, body mass index; PSQI, Pittsburgh Sleep Quality Index 95% CI, 95% confidence interval; OR, Odds ratio; OSA, obstructive sleep apnea; RR, relative risk.

Sleep was evaluated by different assessment measures, most of them subjective – Pittsburgh sleep quality index (*n* = 9), Epworth sleepiness scale (*n* = 4), STOP-Bang questionnaires (1), insomnia severity index (*n* = 1), Athens insomnia scale (*n* = 4), sleep scale (*n* = 1), Jenkins sleep scale (JSS-4) (*n* = 1), Berlin questionnaire (*n* = 1) and questions (*n* = 3), such as “Have you had problems with sleep recently?” “Have you had any trouble sleeping recently?” and “Normally, how many hours do you sleep?” Four studies used objective measurements: actigraphy (*n* = 2) and polysomnography (*n* = 2). Three studies used a single self-reported question to assess sleep (11, 23, 27) and only two made combined use of objective and subjective measures: the Pittsburgh sleep quality index (PSQI), Epworth sleepiness scale, actigraphy and polysomnography (20, 29) (Table 1).

Prevalence of insomnia ranged from 0.8 to 46.5% (21, 26); of poor sleep quality (IQSP >5), from 40.7 to 65.9% (30); of short sleep duration (5 h), from 11 to 25.5% (20, 23); and of long sleep duration (9 h), from 4.7 to 38.5% (10, 11). Another study, using a different cutoff point for sleep duration, revealed 13.4% prevalence of short sleep (< 6 h) and 12.5% of long sleep (> 8 h) (31). A single study estimated a prevalence of 8.9% for severe sleep apnoea, 16.6% for poor sleep efficiency and 9.2% for prolonged latency (20) at baseline (Table 1). Also, 32% of older men and 40% of women had sleep disorders (9).

Frailty was identified using mainly the Fried frailty phenotype (*n* = 12), frailty index (*n* = 2), Tilburg frailty indicator (*n* = 2), frail scale (1), Groningen frailty indicator (*n* = 1), Kihon checklist and comprehensive frailty assessment tool (*n* = 1). Note, however, that more than half the studies (66.7%) used the Fried frailty phenotype (9–12, 20, 22, 24, 27–31) (Table 1).

Studies using the Fried frailty phenotype found prevalence of pre-frailty ranging from 24.1% (10) to 53.8% (11), and frailty prevalence of 9.2% (11) to 41.2% (28). A single study, which evaluated frailty by other methods, pointed to prevalence of frailty of 19.6% (frailty index), 31.2% (Tilburg frailty indicator), 30.6% (Groninger frailty indicator) (30) (Table 1). The only two studies assessing incidence of pre-frailty found 35.8% (21) and 50.5% (20). Incidence of frailty was found to be 14.4% (20), 30.0% (21), 30.4% (22) and 33.2% (23).

After adjustments, one cross-sectional study carried out with 1,643 older adults Mexicans revealed that the high risk of OSA associated with frailty (OR = 1.59; 95% CI = 1.07–2.38) in the global sample and among women (OR = 1.84; 95% CI = 1.05–3.22), on the contrary, among men there was no association (OR = 1.65; 95% CI = 0.65–2.19) (11).

Excessive sleepiness was evaluated in five studies evaluated excessive sleepiness and only one cross-sectional study observed its association with frailty (OR = 3.67, 95% CI = 2.03–6.61) (28).

One cross-sectional study revealed that reduced sleep efficiency (OR = 1.37; 95% CI = 1.12–1.67) and sleep latency (OR = 1.42; 95% CI = 1.10–1.82) were associated with frailty (29). On the other hand, a longitudinal analysis of that same population found no association between sleep latency and frailty (OR = 1.20; 95% CI 0.84–1.71), while nighttime wakefulness of 90 min or longer was the only parameter of objective sleep–wake patterns associated whit frailty/dead or dead (OR = 1.31; 95% CI = 1.04–1.66 and 1.57 95% CI = 1.12–2.20 respectively) (20). Poor sleep quality (HEI > 5) was associated with pre-frailty (12, 30) and frailty (9, 24, 29–32), but only in cross-sectional studies. Sleep complaints in older adult women living in rural areas were associated with frailty in a cross-sectional analysis (OR = 3.20; 95% CI = 1.33–7.68) (27), but not in a longitudinal analysis (RR = 1.41; 95% CI = 0.94–2.12) (11).

In the 80-year-old age group, poor sleep quality was independently associated with frailty (OR = 1.78; 95% CI = [1.02, 3.11]) (31). Older adults with a sleep disorder were 0.66 times more likely (OR = 1.66; 95% CI = [1.47—1.87]) to be frail than those with no sleep disorder (*p* < 0.001) (9).

Insomnia was associated with frailty in both cross-sectional analyses (10, 26) and longitudinal analyses (21, 22). Short (< 5 h) sleep duration was associated with frailty in a cross-sectional analysis (OR = 2.67; 95% CI = 1.01–7.08) (10), but not in a longitudinal study (OR: 1.15; 95% CI = 0.82–1.62) (20). Long (9 h) sleep duration was associated with frailty, but only in cross-sectional analyses (12, 23, 32).

Methodological quality evaluation of 14 cross-sectional studies classified only one as being of low quality (Supplementary Table S1), while none of the four cohort studies was found to be of low quality (Supplementary Table S2).

## 4 Discussion

The 17 studies included in this systematic review found that insomnia, short and long sleep duration, poor efficiency, prolonged latency, obstructive sleep apnoea and poor sleep quality are associated with pre-frailty and frailty in community-dwelling older adults in several different countries.

A variety of methods for assessing exposure and outcome were identified. On the one hand, this reflects the complexity of evaluating both sleep and frailty and, on the other, it indicates the need to standardize methods for evaluating these events. Use of a single self-reported question to assess sleep may fail to consider other sleep problems.

In that light, use of a combination of objective and subjective measures of sleep can yield more robust results. Nonetheless, this review found that studies using objective measures of sleep are scarce and, in longitudinal analysis, no association was observed between the sleep parameters evaluated and frailty. This reinforces the need for further longitudinal studies using objective sleep assessment measures.

Methods used assess frailty differed in the dimensions evaluated, reflecting the multidimensionality of this condition. The frailty phenotype, for example, evaluates five criteria in the physical dimension (fatigue, involuntary weight loss, muscle weakness, gait speed and physical activity) and individuals can be classified on these criteria as robust, pre-frail and frail (7). Other approaches address non-physical (e.g., cognitive and clinical) dimensions. However, these approaches are not always easy to apply in clinical practice and epidemiological research, as they can be time-consuming and entail broader evaluation.

Of the 17 studies evaluated, 16 found independent associations between sleep problems and frailty. As regards mechanisms for this association, one hypothesis is that alterations in sleep may cause hormonal changes, such as decreases in growth hormone, insulin-like growth factor and testosterone production, with impacts including increased muscle protein breakdown, which favors the emergence of geriatric syndromes (sarcopoenia and frailty) (33). This occurs with sleep disorders that shorten the deep sleep phase (34), which also results in increased cortisol levels, culminating in impaired physical function (33).

Given that inflammation is known to be one of the pathophysiological bases of frailty (7), the association between sleep quality and frailty stages could also be explained on the basis that sleep disorders, such as insomnia and long sleep duration, favor changes in inflammation levels involving increased C-reactive protein, interleukin-6 and tumor necrosis factor (TNF-*α*) (32, 35, 36). In addition, long-duration sleep may result from sleep disorders such as insomnia, obstructive sleep apnoea and restless leg syndrome (37). Also, short-duration sleep can also result in lowered testosterone levels and increased oxidative stress (33), thus contributing to the development of frailty.

Among the studies that showed significant associations between sleep disorders and frailty (*n* = 17), almost all (*n* = 13) performed adjustments for confounding variables and were adjusted for sex and age.

As age progresses, hormonal changes, such as a gradual reduction of free testosterone, occur in both sexes, culminating in greater susceptibility to frailty in men and sarcopenia in women (35, 38, 39). Sarcopenia, in turn, is part of the pathophysiological basis of frailty. Also, unlike men, women with over 5 years of menopause or early menopause, and thus falling estrogen levels, are at higher risk of musculoskeletal disorders, such as sarcopenia, which can progress to frailty (40). Common changes in aging include reduced resting energy expenditure in skeletal muscle. This decline occurs faster in females, suggesting that women are more prone to developing frailty (38, 41, 42). Since the evidence above indicates important differences between the sexes, studies that stratify by sex are important in order to understand better how the association between sleep and frailty behaves in men and women.

It is known that sleep problems may occur in older adults due to the presence of comorbidities, thus increasing their susceptibility to frailty (20). This underlines the need to consider these conditions in the process of adjustment for confounding variables in statistical analysis. Ten of the studies included in this review performed adjustments for comorbidities.

However, no pattern was observed among the studies in the selection of confounding variables for adjustment, which partly undermines the degree to which their findings approximate to reality.

Furthermore, a study included in this review evaluated the bidirectional relationship between frailty and insomnia and concluded that the association between frailty and insomnia was significant, especially in men (21), which highlights the need for future studies on the causal relationship between frailty and sleep quality and duration.

Our results also point to the need for more comprehensive assessment of sleep in older adults, including screening, diagnosis and intervention measures to improve sleep quality, mainly because several aspects of sleep quality are associated with physical frailty in this age group. Most health care services for frail older adults do not currently screen for and treat sleep disorders. Given the importance of frailty in current geriatric medicine, it is important to perform a comprehensive geriatric evaluation in older patients with sleep disorders.

### 4.1 Strengths and limitations

The strengths of this review include the fact that it included 17 studies on the subject in different countries, most of which were of moderate to high methodological quality. The relationship between sleep issues and frailty has only recently been investigated and, considering the impact that poor sleep quality and frailty can have on the health of older adults, as well as the accelerating growth of older adult populations and the reversibility pre-frailty and frailty, it is important to investigate the association between sleep quality and frailty.

On the other hand, most of the studies were cross-sectional, posing a need for more cohort-type studies, which would afford a better understanding of the causal relationship between sleep quality and frailty conditions. Moreover, the studies reviewed may be prone to recall bias, specifically in self-reported sleep variables. Furthermore, a meta-analysis was not possible to be carried out due to the heterogeneity of the studies included in this review, for example, different ways of assessing sleep quality and frailty. Another limitation is the fact that this systematic review included only articles published in English, Spanish and Portuguese, which may limit the representation of some regions of the world, especially those areas whose studies are published in other languages.

## 5 Conclusion

We observed that poor sleep quality (insomnia, sleep deprivation, obstructive sleep apnea, excessive daytime sleepiness) and short/long sleep duration was associated with pre-frailty and frailty in older adults. This combination may result in other adverse health outcomes compromising the quality of life of this population.

The results of this review also suggest a need for further research on the relationship between sleep and frailty, including longitudinal studies and surveys stratified by sex.

## Data Availability

The original contributions presented in the study are included in the article/Supplementary material, further inquiries can be directed to the corresponding author.
