# Supplementary material for: Sleep quality and duration and frailty in older adults: a systematic review
Source: Front Public Health. 2025 Feb 26;13:1539849. doi: 10.3389/fpubh.2025.1539849 (PMC11898741; doi:10.3389/fpubh.2025.1539849)
Supplement: Supplementary file 1 [file Supplementary_file_1.docx]

Supplementary Material

# Supplementary Data

Table S1 - Assessment of methodological quality according to the checklist proposed by Joanna Briggs Institute for cross-sectional studies.

| Auth orYears | 1.Criteria for inclusion in the sample? | 2. Study subjects and the setting described? | 3.  Exposure measured in a valid and reliable way? | 4. Objective, standard criteria used for measurement of the condition | 5.Confounding factors identified? | 6. Strategies to deal with confounding factors stated? | 7. Outcomes measured in a valid and reliable way? | 8.Appropriate statistical analysis use? | Overall appraiasal |
| --- | --- | --- | --- | --- | --- | --- | --- | --- | --- |
| Vaz-Fragoso et al., 2009 | Yes | Yes | Yes | Yes | Yes | Yes | Unclear | Unclear | High |
| Ensrud et al., 2009 | Yes | Yes | Yes | Yes | Yes | Yes | Yes | Yes | High |
| Moreno-Tomayo et al 2017 | Yes | Yes | Unclear | Yes | Unclear | Unclear | Yes | Yes | Moderate |
| Sun et al., 2020 | Yes | Yes | Yes | Yes | Yes | Yes | Yes | Yes | High |
| Moreno-Tomayo et al., 2020 | Yes | Yes | Yes | Yes | Yes | Yes | Yes | Yes | High |
| Shih et al., 2020 | Yes | Yes | Yes | Yes | Yes | Yes | Unclear | Yes | High |
| Alqatani et al.,2021 | Yes | Yes | Yes | Yes | Yes | Yes | Unclear | Unclear | High |
| Balomenos et al., 2021 | Not | Unclear | Yes | Yes | Yes | Yes | Yes | Yes | High |
| Moreno-Tomayo^b^ et al., 2021 | Yes | Yes | Yes | Yes | Unclear | Yes | Yes | Yes | High |
| Yu et al.,2022 | Yes | Unclear | Yes | Yes | Not | Unclear | Unclear | Unclear | Low |
| Fan et al., 2022 | Yes | Yes | Yes | Yes | Yes | Yes | Unclear | Unclear | High |
| Aditi et al 2023  India | Yes | Yes | Yes | Yes | Unclear | Yes | Yes | Yes | High |
| Mizuno et al 2023  Japan | Yes | Yes | Yes | Yes | Yes | Yes | Yes | Yes | High |

Table S2 - Assessment of methodological quality according to the checklist proposed by Joanna Briggs Institute for cohort studies.

|  | - Ensrud et al., 2012 | - Nemoto et al 2020 | - Moreno-Tomayo^a^ et al., 2021 | - Chen et al., 2022 |
| --- | --- | --- | --- | --- |
| 1-The two groups similar and recruited from the same population? | - Yes | - Yes | - Yes | - Yes |
| 2- The exposures measured similarly to assign people to both exposed and unexposed groups? | - Yes | - Yes | - Yes | - Yes |
| 3- Exposure was measured validly and reliably? | - Yes | - Yes | - Yes | - Yes |
| 4- Confounding factors were identified? | - Yes | - Yes | - Yes | - Unclear |
| 5- Strategies for dealing with confounding factors were stated? | - Yes | - Unclear | - Yes | - Yes |
| 6- Were the groups/participants free from the outcome at the beginning of the study (or at the time of exposure)? | - Yes | - Yes | - Yes | - Yes |
| 7- Results were measured validly and reliably? | - Yes | - Yes | - Yes | - Yes |
| 8- Follow-up time was reported to be long enough for results to occur? | - Unclear | - Yes | - Unclear | - Yes |
| 9- Follow-up was complete and, if not, reasons for loss to follow-up were described and explored? | - Yes | - Unclear | - Yes | - Yes |
| 10- Strategies were used to address incomplete follow-up? | - Yes | - Unclear | - Unclear | - Yes |
| 11- Strategies to address incomplete follow up utilized? | - Yes | - Unclear | - Yes | - Yes |
| Overall appraiasal | - High | - Moderate | - High | - High |

**
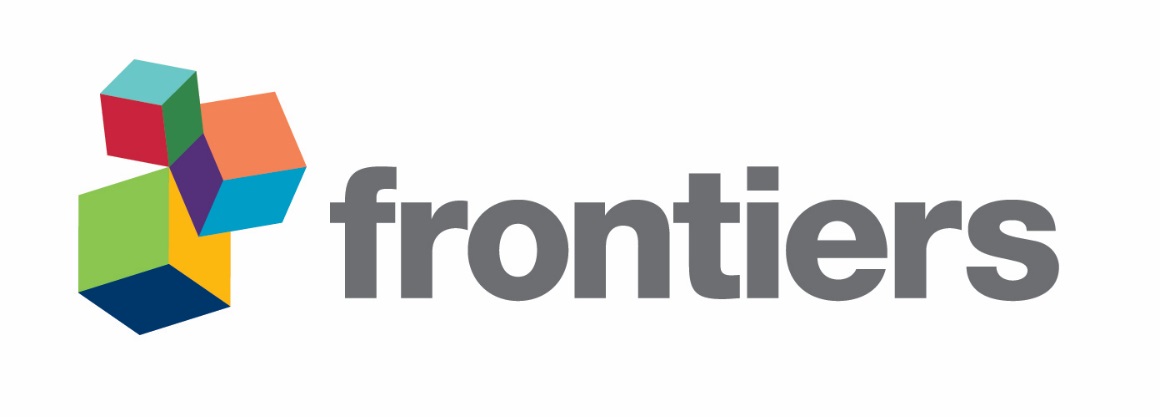
**
